# Sociodemographic and biochemical correlates of self-reported taste dysfunction: an NHANES analysis

**DOI:** 10.1515/med-2026-1496

**Published:** 2026-08-24

**Authors:** Dhruva Arcot, Subhankar Chakraborty

**Affiliations:** Willard Grizzell Middle School, Dublin, OH, USA; Division of Gastroenterology, Hepatology and Nutrition, The Ohio State University Wexner Medical Center, Columbus, OH, USA; Department of Internal Medicine, The Ohio State University Wexner Medical Center, Columbus, OH, USA

**Keywords:** taste problems, NHANES, biochemical markers, fatty acids, albumin, copper

## Abstract

**Objective:**

Taste dysfunction affects approximately 1 in 5 Americans over age 40, yet its relationship with biochemical and hematological markers remains poorly understood. This study investigated whether biochemical and hematologic markers are associated with self-reported taste dysfunction.

**Methods:**

Pooled NHANES data from 2011 to 2014 were analyzed. The final sample included 3,595 adults aged ≥20 years who provided a definitive response regarding whether they experienced taste problems. Survey-weighted logistic regression identified independent predictors among socio-demographic factors and biochemical markers.

**Results:**

219 (6.1 %) individuals reported taste problems. In fully adjusted models, advancing age (aOR=1.02; p=0.034), Non-Hispanic White (aOR=2.54) or Black race (aOR=2.45), less than a high school education (aOR=1.59), and lower household income (p=0.002) were independently associated with taste dysfunction. Only serum albumin (aOR=0.41; p=0.044), percent of saturated fatty acids (aOR=0.88; p=0.006), percent of monounsaturated fatty acids (aOR=1.14; p=0.006), and serum copper levels (aOR=1.02; p=0.006) were associated with taste problems. None of the hematologic labs examined were associated with taste problems.

**Conclusions:**

Sociodemographic factors and limited biochemical abnormalities are associated with self-reported taste problems. Future prospective studies are required to confirm these findings.

## Introduction and background

Roughly 1 in 5 Americans over the age of 40 have reported some alteration in their taste, according to the National Institutes of Health (NIH) [1]. The different kinds of taste alteration can be classified into complete and partial. Partial taste alteration is much more common than complete. The 2 types of partial taste alteration are dysgeusia and hypogeusia. Dysgeusia is a condition where a foul, metallic, or rancid taste occurs in the mouth [2], 3]. Around 1 in 20 Americans report dysgeusia, and most of these cases are reported by women (64 %) [2], 3]. Hypogeusia is a condition where people experience a reduced sense of taste. This condition is rare compared to dysgeusia. An extreme case of taste alteration is complete taste loss. This is also known as ageusia. Ageusia is the least common when compared to other taste conditions. About 1 or 2 in 1,000 people experience ageusia (0.001 %) [1], [3], [4], [5], [6].

The tongue plays an important role in picking up and transmitting taste sensations to the brain. It picks up 5 basic flavors, which are sweet, sour, salty, bitter, and savory, or “umami,” using receptors called taste buds to transmit these flavors to the brain. The brain then interprets and assigns flavors to these. There is a common myth that certain parts of the tongue pick up certain tastes more than others, but surprisingly, this is not true. All taste receptors across the tongue interpret all flavors. However, the sides and tip of our tongue are generally more sensitive to taste than the middle [7], 8].

Diagnoses with any form of taste loss are usually related to damage or impairment of nerves related to the pathway of taste from the tongue to the brain. The cranial nerves transmit signals from the tongue to the brain. They include the facial nerve, vagus nerve, and the glossopharyngeal nerve. The facial nerve is responsible for transmitting taste sensations from the anterior portion of the tongue, while the glossopharyngeal nerve transmits the sensation of taste from the posterior portion of the tongue. The vagus nerve is responsible for the taste sensations in the very back of the mouth, known as the epiglottis [7].

There may be many factors contributing to alterations in taste. Some of the most common factors are respiratory and ear infections, poor oral hygiene, dental problems, exposure to chemicals such as insecticides certain medications, and head injury [9]. One of the most common causes of taste problems is a loss of olfactory sensation. According to an article from the American Academy of Family Practice, 95 % of taste disorders are caused by an impairment of smell [3]. This is because the human body’s taste and smell are linked through chemosensation, the body system used to sense chemicals. When eating food, the molecules of food reach the back of the nose and interact with olfactory receptors (termed retro-nasal olfaction) [10]. This is why smell plays such a big part in taste. The remaining 5 % of taste issues are likely multifactorial in nature. One of the potential mechanisms underlying taste problems is biochemical or hematologic abnormalities, which is the focus of our research paper.

Biochemical markers are organic molecules like proteins, enzymes, electrolytes, lipids, micronutrients, inflammatory markers, or hormones found in blood, body fluids, or tissues that, when perturbed, can create an abnormality in biological processes. Hematological markers are measurements related to blood cells and their properties. Research on the relationship between biochemical or hematological markers, and loss of taste is limited. A study from 2021 described the relationship between patients diagnosed with COVID-19-related taste impairments and biochemical abnormalities. This study had patients rate their taste sensation on a visual analogue scale from 0 to 10, where a score of 0 indicated complete loss of taste (ageusia), while a score of 10 meant that there were no impairments to taste. Compared to patients who had normal taste, those with any degree of taste loss (VAS score<10) had a significantly lower neutrophil count and a higher lymphocyte count [11].

The relationship between biochemical markers and the taste loss is not fully understood. A handful of biochemical factors and their association with taste problems have been reported, but large scale epidemiologic studies supporting a cause-effect relationship are lacking. One vitamin that has shown a clear correlation with taste dysfunction is vitamin B12. The epithelial cells are responsible for being a protective cover for the body. A deficiency of vitamin B12 can cause a disruption in these cells, which in turn can lead to tongue pain, redness, and reduction in the number of papillae [12]. Some of the other biochemical taste markers that have been reported to contribute to taste loss include zinc and iron [13].

Examining the relationship between biochemical and hematological markers, and taste impairment can be immensely beneficial to the prevention and treatment of taste disorders. To address the gap in knowledge about how these markers may be associated with taste dysfunction, we conducted the current study, where we used a publicly available nationally representative database of participants who were asked a question about taste problems in the past year. We compared a large group of biochemical and hematological markers between those who did vs. those who did not experience taste problems in the past one year to identify potential associations. By exploring this area, our research could aid in the treatment and prevention of taste disorders.

## Methodology

**Figure 1:**
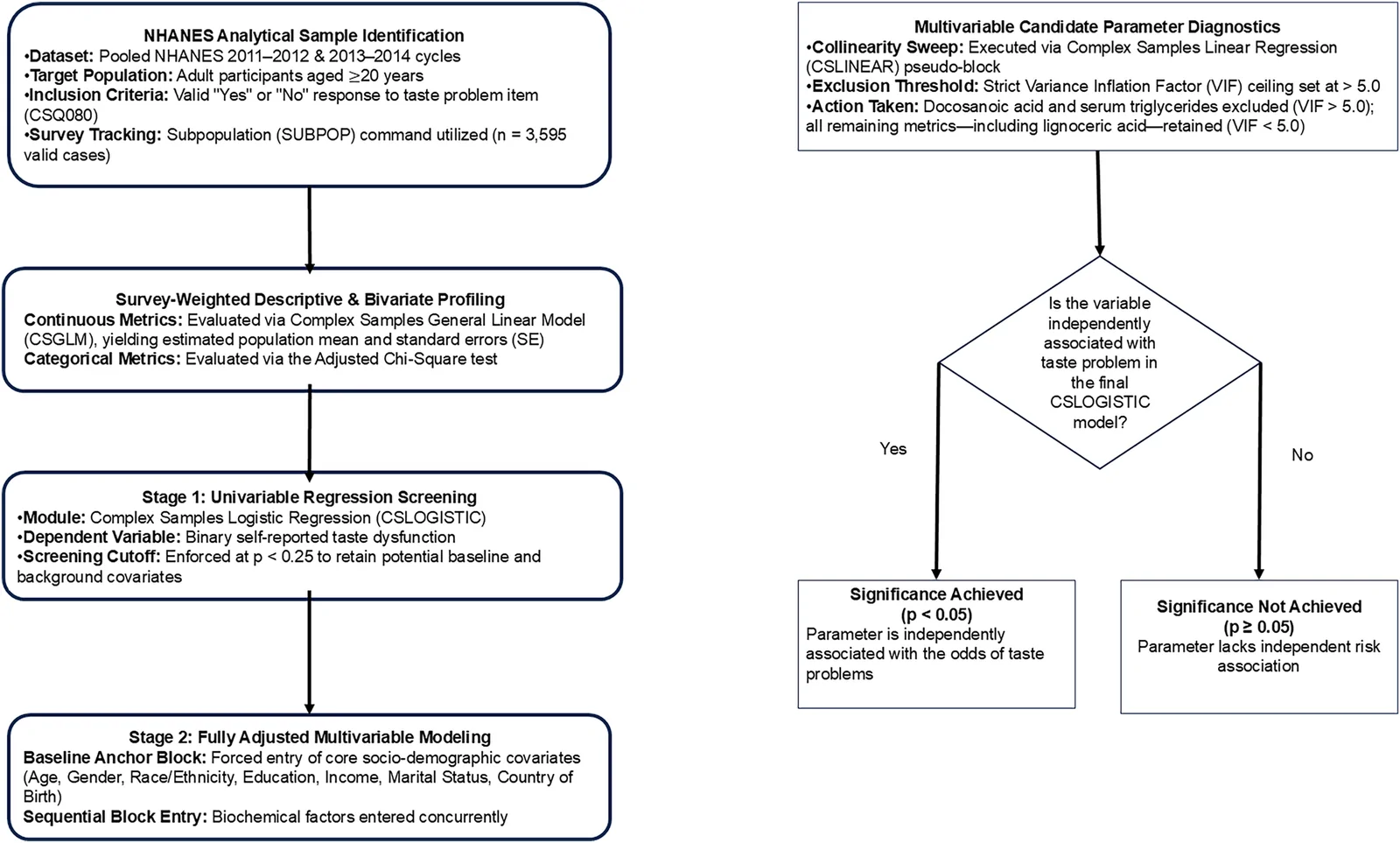
NHANES analytic workflow for identifying independent correlates of self-reported taste dysfunction. This flowchart outlines the sequential analytic procedures used to derive the final survey-weighted logistic regression model. Adult participants (≥20 years) from the 2011–2012 and 2013–2014 NHANES cycles were included if they provided a valid “Yes/No” response to the taste problem item (CSQ080), yielding a subpopulation of 3,595 cases. Descriptive and bivariate profiling was conducted using complex-sample general linear models for continuous variables and adjusted chi-square tests for categorical variables. Univariable logistic regression screening (p<0.25) identified candidate covariates for multivariable modeling. The fully adjusted model incorporated a forced baseline block of socio-demographic variables (age, gender, race/ethnicity, education, income, marital status, and country of birth), followed by sequential entry of biochemical factors. Collinearity diagnostics were performed using complex-sample linear regression, applying a strict VIF>5.0 exclusion threshold. Final model decisions were based on whether each parameter demonstrated independent significance (p<0.05), identifying variables with true associations.

The purpose of the study was to investigate whether biochemical or hematological markers are associated with taste problems. Figure 1 above shows the steps used in the data analysis for this study.

## Data source and target population

We used cross-sectional data from the National Health and Nutrition Examination Survey (NHANES), a nationally representative multi-stage probability sample of the non-institutionalized civilian United States population conducted by the National Center for Health Statistics (NCHS). NHANES protocols were approved by the NCHS Research Ethics Review Board, and all participants provided written informed consent. Data from two consecutive survey cycles (2011–2012 and 2013–2014) were pooled for this analysis because these were the two cycles where the participants were asked questions about taste problems.

Inclusion criteria included adult participants aged 20 years or older who provided a valid, definitive “Yes” or “No” response to the primary chemosensory survey item (CSQ080): *“Have you had a problem with taste in the past 12 months?”* Out of 24,537 initial interviewees, after excluding those with missing responses, 3,595 valid cases remained.

## Outcomes and covariate domain categorization

The primary outcome was binary and was self-reported taste dysfunction within the preceding 12 months. Candidate independent predictors and covariates were systematically categorized *a priori* into two functional domains:

– **Socio-demographic factors (anchor block):** These comprised participant age at screening (continuous variable), biological gender, race/ethnicity (categorical variable), educational attainment (categorical variable), annual household income (categorical variable), marital status (categorical variable), and country of birth (categorical variable).
– **Nutritional and biomarker indices:** These comprised serum levels of various biochemical markers, including fatty acids, liver and kidney function markers, hematologic indices, and nutritional/vitamin metrics.

## Statistical analysis and design weighting

All statistical procedures were performed using the Complex Samples module in IBM SPSS Statistics (Version 28). Analyses incorporated Mobile Examination Center sample weights (WTMEC2YR), masked variance pseudo-strata, and primary sampling units (PSUs). Missing data and age-based exclusions were handled using the Complex Samples subpopulation (SUBPOP) command, which restricts estimation to the analytic subpopulation while retaining the full survey design structure. This approach prevents distortion of population variance and avoids the artificially reduced standard errors that occur when cases are removed through row-wise deletion. 

## Survey-weighted descriptive profiling

Because biochemical and nutritional metrics typically exhibit non-normal distributions in human populations, population characteristics were generated using survey-weighted procedures. Continuous parameters were modeled via the Complex Samples General Linear Model (CSGLM) and reported as estimated marginal means with standard errors (SE). Categorical parameters were converted to population-weighted frequencies expressed as percentages, with group differences evaluated using the Rao-Scott adjusted Chi-Square test.

## Purposeful variable selection framework

A structured, step-wise model-building framework was applied to identify independent biomarkers of taste dysfunction:

– **Stage 1: univariable regression screening:** Individual candidate biomarkers were initially evaluated for association with taste dysfunction using univariate Complex Samples Logistic Regression (CSLOGISTIC) models. To prevent the premature exclusion of clinically relevant background markers, a relaxed significance threshold of p<0.25 was used to qualify candidates for multivariable consideration.
– **Stage 2: multicollinearity diagnostics for related continuous variables:** To evaluate multicollinearity among conceptually related biochemical markers (e.g., liver enzymes, kidney function indices, or serum fatty acids), all variables within each marker group were entered simultaneously into a Complex Samples Linear Regression (CSLINEAR) model. Collinearity was assessed using the Variance Inflation Factor (VIF), with values >5.0 indicating substantial multicollinearity and resulting in exclusion from further modeling. Variables that also demonstrated weak contribution in the collinearity model (p>0.25) were removed. For serum fatty acids, several candidates met univariable significance criteria and required additional data transformations, as described below.
– **Transformation of fatty acid components:** Fatty acids that demonstrated statistical significance in the univariate logistic regression screening were further evaluated for multicollinearity. Several of these candidate fatty acids showed severe collinearity (VIF>10), so they were first aggregated into biologically coherent families – saturated fatty acids (SFAs), monounsaturated fatty acids (MUFAs), and omega-3 fatty acids – by summing their individual concentrations. When reassessed, substantial collinearity persisted between the SFA and MUFA groups. To resolve this, each fatty-acid family was converted into a proportional measure relative to the total fatty-acid pool.

For example:

$$\text{SFA proportion}=\frac{\text{SFA}\;\text{level}}{\text{SFA}+\text{MUFA}+\text{Omega}-3\;\text{levels}}\text{.}$$

After applying these proportional transformations, collinearity diagnostics indicated that the three fatty-acid groups no longer exhibited meaningful multicollinearity (VIF<1.1).

## Multivariate regression

The final multivariable models were constructed using a sequential block entry approach:

1. **The socio-demographic baseline anchor block:** (Age, gender, race/ethnicity, education, income, marital status, and country of birth) was forced into a single “baseline” model to handle background confounding.
2. **Laboratory markers:** were entered group-wise into the basic model to evaluate separately the impact of each group of biochemical markers on the taste problem.

We employed the Complex Samples Logistic Regression model to perform the multivariate regression. Associations are expressed as Adjusted Odds Ratios (aOR) with corresponding 95 % Confidence Intervals (CI).

## Hypothesis

We hypothesized that there would be differences in hematologic and biochemical markers in people with and without taste dysfunction, and these markers would be independently associated with taste problems after adjusting for demographic characteristics.

## Results

Out of 24,537 interviewees from the pooled NHANES 2011–2014 cycles, the final survey-weighted analytical sample included 3,595 adults (14.7 %) aged 20 years or older who provided a definitive “Yes” or “No” response to the taste problem question. Of these, 219 (6.1 %) reported taste problems, and 3,376 (93.9 %) did not.

There were significant demographic differences between the two groups based on age, educational attainment, and country of birth (Table 1). Participants who reported taste problems were older than those without taste problems (mean age 60.3 years± 1.2 vs. 57.5 years ± 0.3; p=0.025).

**Table 1:** Demographic and socioeconomic characteristics of US adults stratified by 12-month self-reported taste dysfunction: NHANES 2011–2014.

Demographic variable	With taste problems (n=219)	Without taste problems (n=3,376)	p-Value
**Age (years at screening)**, mean (SE)	60.3 (1.2)	57.5 (0.3)	**0.025**
**Gender**			0.282
Male	46.8 %	47.2 %	
Female	53.2 %	52.8 %	
**Race/hispanic origin**			0.070
Mexican American	4.9 %	5.4 %	
Other Hispanic	4.4 %	5.6 %	
Non-Hispanic White	74.4 %	71.3 %	
Non-Hispanic Black	12.9 %	10.6 %	
Non-Hispanic Asian	2.6 %	4.7 %	
Other/multi-racial	0.8 %	2.4 %	
**Educational attainment (adults 20+)**			**0.026**
Less than 9th grade	8.5 %	6.8 %	
9th–11th grade (no HS diploma)	17.4 %	11.1 %	
High school graduate/GED	17.8 %	21.7 %	
Some college/associate degree	27.5 %	29.5 %	
College graduate or above	28.5 %	30.8 %	
**Annual household income**			0.052
Low income (<$20,000)	22.4 %	15.2 %	
Lower-middle ($20,000–$44,999)	36.4 %	27.0 %	
Middle ($45,000–$74,999)	12.0 %	20.3 %	
High income (≥$75,000)	26.6 %	34.4 %	
**Marital status**			0.603
Married or living with a partner	60.6 %	65.2 %	
Previously married	28.6 %	25.9 %	
Never married	10.8 %	9.0 %	
**Country of birth**			**0.022**
United States	88.6 %	83.9 %	
Outside United States/other	11.0 %	16.0 %	

Pooled NHANES 2011–2014 cycles. Row percentages represent population-weighted distributions within each demographic category and sum to 100 % across the “With” and “Without” taste-problem groups. Categorical estimates were generated using Complex Samples cross-tabulations (CSCROSSTABS) incorporating Mobile Examination Center sample weights (WTMEC2YR), masked variance pseudo-strata, and primary sampling units. Continuous variables were evaluated using Complex Samples General Linear Models (CSGLM) and are presented as design-adjusted estimated marginal means with standard errors (SE). Boldface indicates statistical significance at p<0.05. Values in bold indicate those that were statistically significant.

Regarding educational attainment, the proportion of those who did not complete high school was higher among those with taste problems (25.9 % vs. 17.9 %, p=0.026). There was also a higher proportion of individuals born within the US among those with taste problems compared to those without (88.6 % vs. 83.9 %, p=0.022).

The proportion of households with annual income less than $75,000 was higher among the taste problems group (70.8 % vs. 62.5 %), but it did not reach statistical significance. There was no difference between the groups in the proportion of females or by race.

## Laboratory biomarkers and taste problems

Weighted comparisons of estimated marginal means (EMM) for laboratory biomarkers revealed several statistically significant variations across metabolic profiles, environmental trace minerals, serum vitamin co-factors, and circulating fatty acid speciation profiles between individuals with and without taste problems.

## Metabolic, liver, and kidney biomarkers

Participants with taste problems had lower serum albumin (4.15 g/dL vs. 4.27 g/dL, p=0.013) but higher alkaline phosphatase (75.5 IU/L vs. 68.0 IU/L; p=0.009), creatinine (0.99 mg/dL vs. 0.91 mg/dL; p=0.030), and lactate dehydrogenase (LDH) (135.9 IU/L vs. 129.5 IU/L; p=0.042). Conversely, serum chloride levels were lower in those with taste problems (102.9 mmol/L vs. 103.8 mmol/L; p=0.008). No differences were observed for other liver or kidney biochemical markers (Supplementary Table 1).

### Environmental heavy metals and trace minerals

Among the analyzed trace minerals and heavy metals, serum copper was the only one that was significantly different between the two groups (p=0.046), with higher concentrations in individuals reporting taste problems (124.3 µg/dL, SE=3.6) than in those without (116.6 µg/dL, SE=1.4). Serum iron levels were lower in those with taste problems (79.9 µg/dL vs. 87.5 µg/dL), but this was not significant (p=0.065). Other minerals and heavy metal levels did not differ between the two groups (Supplementary Table 1).

### Circulating serum lipid levels and hematologic indices

No statistically significant differences were detected across circulating serum lipid profiles (total and direct cholesterol, LDL cholesterol and triglycerides) or hematologic indices (total and differential white blood cell count, red blood cell count, hemoglobin, red blood cell indices and platelet count) (Supplementary Table 1).

## Vitamins

Red blood cell (RBC) folate levels were higher in individuals presenting with taste problems (620.9 ng/mL vs. 546.7 ng/mL; p=0.038). Other folate speciation profiles (e.g., 5-Methyl-tetrahydrofolate), vitamin B12, and vitamin D markers (25OHD_2_ and 25OHD_3_), however were similar (Supplementary Table 1).

## Serum fatty acids

Several fatty acids were differentially altered between the two cohorts. Among saturated fatty acids, pentadecanoic acid (C15:0) was elevated (31.1 µmol/L vs. 26.6 µmol/L; p=0.035) while longer-chain saturated fatty acid levels including those of docosanoic acid (C22:0; 64.7 µmol/L vs. 70.8 µmol/L; p=0.030) and lignoceric acid (C24:0; 54.2 µmol/L vs. 60.2 µmol/L; p=0.022) were significantly lower in those with taste problems. Among unsaturated fatty acids, oleic acid (18:1n-9) and docosapentaenoic acid (22:5n-3) levels were higher in the group with taste problems, but neither reached statistical significance (p=0.070 for both). The remaining fatty acid showed no significant variation between groups (Supplementary Table 1).

## Univariate logistic regression

### Socio-demographic factors

In the unadjusted models, several socio-demographic characteristics were significantly associated with dysgeusia at the prespecified level of significance (p<0.25). This included age, which demonstrated a positive association, with each one-year increase at screening yielding a 2 % increase in the odds of taste dysfunction (OR=1.020, 95 % CI: 1.004–1.036; p=0.019). Race was also significantly associated with taste problems (p=0.008), driven primarily by elevated odds among Non-Hispanic Black (OR=2.660, 95 % CI: 1.400–5.070) and Non-Hispanic White (OR=2.230, 95 % CI: 1.060–4.690) sub-populations relative to the Non-Hispanic Asian group that served as the reference category.

Other demographic factors that were associated with taste problems included the level of educational attainment (p=0.039) and the annual household income (p=0.025). Notably, individuals with a low income (<$20,000) exhibited nearly twice the odds of taste dysfunction compared to those in the high-income bracket (OR=1.910, 95 % CI: 1.050–3.480). Furthermore, being born inside the United States significantly increased the unadjusted odds of reporting taste dysfunction by 53 % (OR=1.530, 95 % CI: 1.080–2.160; p=0.019).

### Metabolic serum biomarkers

Univariate screening identified multiple metabolic, liver, and renal biomarkers associated with taste dysfunction. Serum albumin levels demonstrated an inverse relationship (OR=0.310, 95 % CI: 0.130–0.730; p=0.010) while ALT (OR=1.004, p=0.021), AST (OR=1.006, p=0.032), creatinine (OR=1.340, p=0.011), serum glucose (OR=1.010, p=0.034), and LDH (OR=1.010, p=0.016) showed a positive association with taste problems.

Serum potassium (OR=1.460, p=0.170) and bicarbonate (OR=1.060, p=0.210) were positively associated, while sodium (OR=0.950, p=0.150) and chloride (OR=0.930, p=0.008) levels were negatively associated with taste dysfunction.

Several other biochemical factors were significant at the preset level of significance (p<0.25) and are summarized in Table 2.

**Table 2:** Univariate binomial logistic regression analysis of factors associated with 12-month self-reported taste dysfunction (p<0.25).

Independent predictor variable	Odds ratio (95 % CI)	p-Value
**Socio-demographic characteristics**		
Age (per 1-year increase at screening)	1.020 (1.004–1.036)	**0.019**
Race/Hispanic origin	–	**0.008**
Mexican American	2.020 (0.910–4.470)	
Other Hispanic	1.690 (0.810–3.530)	
Non-Hispanic White	2.230 (1.060–4.690)	
Non-Hispanic Black	2.660 (1.400–5.070)	
Non-Hispanic Asian/other	1.000 (reference)	
Educational attainment (adults 20+)	–	**0.039**
Less than 9th grade	1.350 (0.520–3.550)	
9th–11th grade (no HS diploma)	1.230 (0.410–3.670)	
High school graduate/GED	0.540 (0.150–1.920)	
Some college/associate degree	0.710 (0.260–1.890)	
College graduate or above	1.000 (reference)	
Annual household income	–	**0.025**
Low income (<$20,000)	1.910 (1.050–3.480)	
Lower-middle ($20,000–$44,999)	1.740 (0.900–3.370)	
Middle ($45,000–$74,999)	0.760 (0.380–1.530)	
High income (≥$75,000)	1.000 (reference)	
Country of birth	–	**0.019**
Born inside United States	1.530 (1.080–2.160)	
Born outside United States/other	1.000 (reference)	
**Metabolic serum biomarkers**		
Albumin, g/dL	0.310 (0.130–0.730)	**0.010**
Alanine aminotransferase ALT, IU/L	1.004 (1.001–1.007)	**0.021**
Aspartate aminotransferase AST, IU/L	1.006 (1.001–1.011)	**0.032**
Alkaline phosphatase, IU/L	1.006 (0.990–1.013)	0.080
Bicarbonate, mmol/L	1.060 (0.970–1.160)	0.210
Creatinine, mg/dL	1.340 (1.080–1.670)	**0.011**
Serum glucose, mg/dL	1.010 (1.000–1.010)	**0.034**
Lactate dehydrogenase LDH, IU/L	1.010 (1.002–1.014)	**0.016**
Sodium, mmol/L	0.950 (0.890–1.020)	0.150
Potassium, mmol/L	1.460 (0.840–2.550)	0.170
Chloride, mmol/L	0.930 (0.890–0.980)	**0.008**
**Environmental heavy metals & lipids**		
Blood mercury, total, µg/L	0.950 (0.860–1.040)	0.220
Blood selenium, µg/L	1.010 (1.001–1.010)	**0.010**
Serum copper, µg/dL	1.010 (1.001–1.020)	**0.025**
Iron, refrigerated, µg/dL	0.990 (0.980–1.001)	0.080
Direct HDL-cholesterol, mg/dL	0.980 (0.970–1.004)	0.120
Triglycerides, mg/dL	1.001 (1.000–1.002)	0.250
Total cholesterol, mg/dL	0.990 (0.980–1.003)	0.190
**Hematologic & micronutrient indices**		
White blood cell count (10^3^ cells/µL)	1.060 (0.980–1.140)	0.130
Monocyte number (10^3^ cells/µL)	1.410 (0.930–2.120)	0.090
RBC folate, ng/mL	1.001 (1.000–1.002)	**0.006**
5-methyl-tetrahydrofolate, nmol/L	1.005 (0.990–1.012)	0.170
5-formyl-tetrahydrofolate, nmol/L	0.840 (0.620–1.140)	0.240
**Serum fatty acid profiles**		
Myristic acid (14:0), µmol/L	1.001 (0.990–1.003)	0.170
Pentadecanoic acid (C15:0), µmol/L	1.020 (1.001–1.040)	**0.040**
Palmitic acid (16:0), µmol/L	1.000 (1.000–1.000)	0.140
Margaric acid (C17:0), µmol/L	1.020 (0.990–1.040)	0.100
Docosanoic acid (22:0), µmol/L	0.980 (0.960–1.000)	**0.048**
Lignoceric acid (24:0), µmol/L	0.970 (0.950–0.990)	**0.029**
Myristoleic acid (14:1n-5), µmol/L	1.010 (0.990–1.030)	0.190
Palmitoleic acid (16:1n-7), µmol/L	1.000 (1.000–1.002)	0.160
cis-vaccenic acid (18:1n-7), µmol/L	1.002 (0.990–1.010)	0.160
Oleic acid (18:1n-9), µmol/L	1.000 (1.000–1.000)	0.090
alpha-linolenic acid (18:3n-3), µmol/L	1.002 (0.990–1.010)	0.130
gamma-linolenic acid (18:3n-6), µmol/L	1.010 (0.990–1.020)	0.240
Eicosatrienoic acid (C20:3n-9), µmol/L	1.020 (0.990–1.050)	0.170
Eicosapentaenoic acid (20:5n-3), µmol/L	1.003 (1.000–1.005)	**0.038**
Docosatetraenoic acid (22:4n-6), µmol/L	1.020 (0.990–1.040)	0.090
Docosapentaenoic acid (22:5n-3), µmol/L	1.010 (1.000–1.030)	**0.050**
Docosapentaenoic acid (22:5n-6), µmol/L	1.030 (0.990–1.050)	0.070

All estimates are calculated via complex survey design-adjusted univariate binomial logistic regression models targeting 12-month self-reported taste dysfunction as the binary dependent metric. Models account for population sample weights (WTMEC2YR), pseudo-strata, and primary sampling units. Only predictors meeting the screening threshold of p<0.25 are shown, as these variables were subsequently advanced to multivariable modeling. Odds ratios (OR) reflect the change in odds of reporting taste dysfunction per unit increase in continuous predictors or relative to the designated reference category for categorical predictors. ALT, alanine aminotransferase; AST, aspartate aminotransferase; ALP, alkaline phosphatase; LDH, lactate dehydrogenase; HDL, high-density lipoprotein cholesterol; RBC, red blood cell. Values in bold indicate those that were statistically significant.

### Environmental heavy metals, lipids, and hematologic indices

Among trace minerals and heavy metals, serum selenium (OR=1.010, p=0.010) and serum copper (OR=1.010, p=0.025) were positively associated with taste dysfunction, while mercury (OR=0.950, p=0.220) and iron (OR=0.990, p=0.080) showed inverse associations with dysgeusia.

With respect to lipid profiles, direct HDL-cholesterol (OR=0.980, p=0.120), total cholesterol (OR=0.990, p=0.190), and triglycerides (OR=1.001, p=0.250) showed an inverse relationship with taste problems. Among hematologic and micronutrient indices, white blood cell counts (OR=1.060, p=0.130), monocyte numbers (OR=1.410, p=0.090), 5-methyl-tetrahydrofolate levels (OR=1.005, p=0.170) and RBC folate levels (OR=1.001, 95 % CI: 1.000–1.002; p=0.006) showed a positive association with dysgeusia.

#### Serum fatty acid profiles

Numerous serum fatty acid species met the univariate threshold (p<0.25) for inclusion in further modeling. Within the saturated fatty acids, pentadecanoic acid (C15:0) (OR=1.020, p=0.040), and margaric acid (C17:0) (OR=1.020, p=0.100) showed a positive association while very-long-chain saturated fatty acids like docosanoic acid (22:0; OR=0.980, p=0.048) and lignoceric acid (24:0; OR=0.970, p=0.029) showed a negative association.

Among unsaturated fatty acid profiles, significant positive associations were found for several including eicosapentaenoic acid (20:5n-3; OR=1.003, p=0.038) docosatetraenoic (22:4n-6; p=0.090) and docosapentaenoic acid (22:5n-3; OR=1.010, p=0.050), oleic acid (18:1n-9; p=0.090), myristoleic (p=0.190), palmitoleic (p=0.160), cis-vaccenic (p=0.160), alpha-linolenic (p=0.130), and eicosatrienoic (p=0.170) acids.

## Multivariable logistic regression

### Socio-demographic core profile

We first examined the effect of socio-demographic characteristics- these served as the “core model” to which other groups (e.g. biochemical and hematological marker groups) were added separately. Among these factors, an increase in age was associated with a modest but statistically significant increase in risk, yielding a 2 % increase in the odds of taste dysfunction per additional year at screening (aOR=1.02, 95 % CI: [1.004, 1.04]; p=0.034). Race and Hispanic origin demonstrated robust independent significance within the model (p=0.018). Using Asians and Other Races as the reference cohort, the adjusted odds of reporting taste dysfunction were substantially higher among Non-Hispanic White (aOR=2.54, 95 % CI: [1.15, 5.56]) and Non-Hispanic Black (aOR=2.45, 95 % CI: [1.29, 4.68]) participants.

**Figure 2:**
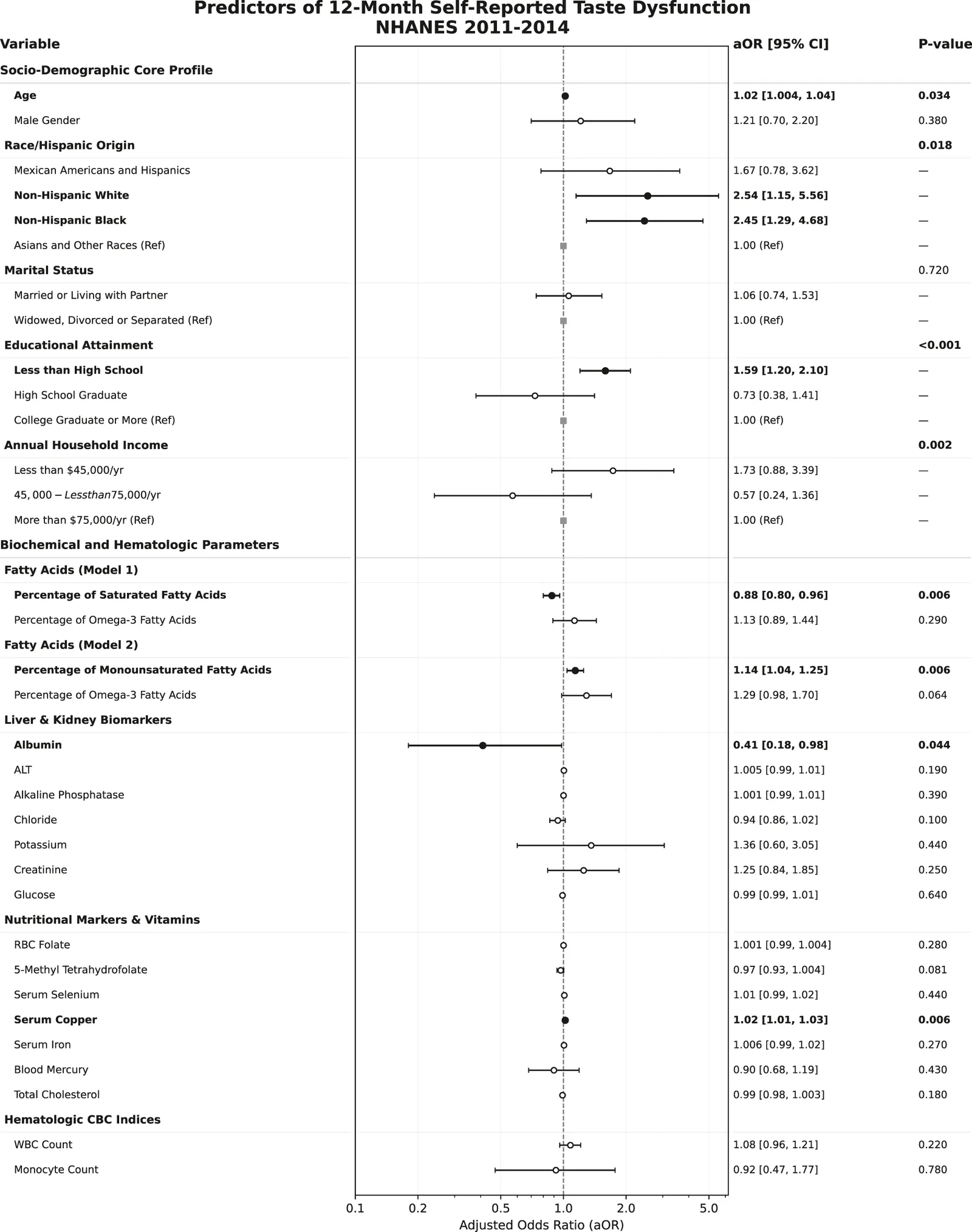
Forest plot of socio-demographic and clinical predictors for self-reported taste dysfunction: The plot displays the adjusted odds ratios (aORs) and corresponding 95 % confidence intervals (CIs) derived from multivariate logistic regression analysis. The vertical dashed line represents the null effect (aOR) = 1.00). Data points to the right of the line indicate increased odds of taste dysfunction, while points to the left indicate decreased odds. The x-axis is presented on a logarithmic scale.Red markers and error bars denote statistically significant predictors (where the 95 % CI does not cross 1.00); blue markers and error bars indicate non-significant predictors. Gray square markers located exactly on the null line represent the designated reference categories (ref) for categorical variables (race/hispanic origin, marital status, educational attainment, and annual household income). Fatty acids (model 1) and fatty acids (model 2) represent separate multivariate models adjusted for the core socio-demographic profile and respective biochemical parameters. *Abbreviations: aOR, adjusted odds ratio; ALT, alanine aminotransferase; CBC, complete blood count; CI, confidence interval; NHANES,* *N* *ational Health and Nutrition Examination Survey; RBC, red blood cell; WBC, white blood cell.*

Educational attainment proved to be highly significant overall (p<0.001), driven primarily by individuals with less than a high school education, who displayed a 59 % increase in the odds of taste dysfunction compared to college graduates (aOR=1.59, 95 % CI: [1.20, 2.10]). Similarly, annual household income was an independent predictor (p=0.002), with lower economic strata (<$45,000/yr) exhibiting a trend toward elevated risk (aOR=1.73, 95 % CI: [0.88, 3.39]) relative to high earners (≥$75,000/yr). Conversely, gender (p=0.380) and marital status (p=0.720) did not significantly influence taste outcomes in the fully adjusted model. Figure 2 illustrates the results of multivariate regression in the form of a Forrest plot.

### Circulating fatty acid profiles (models 1 & 2)

The independent contributions of circulating fatty acid speciation metrics were analyzed using two separate multivariable models to account for collinearity between the saturated and unsaturated fatty acids. In the first model that included the relative percentage of saturated and omega-3 fatty acids, the former demonstrated an inverse association with taste problems; each percentage unit increase in SFAs was associated with a 12 % reduction in the odds of experiencing a taste problem (aOR=0.88, 95 % CI: [0.80, 0.96]; p=0.006).

In the second model, which included the percentage of monounsaturated and omega-3 fatty acids as covariates, the former exhibited a positive association with taste problems- the chances of self-reported taste problems increased by 14 % per unit increase in the percentage of MUFAs (aOR=1.14, 95 % CI: [1.04, 1.25]; p=0.006). The relative percentage of omega-3 fatty acids was not associated with taste problems (Table 3 and Figure 2).

**Table 3:** Multivariate logistic regression analysis of socio-demographic and clinical predictors for 12-month self-reported taste dysfunction: NHANES 2011–2014.

Variable name	Adjusted odds ratio (aOR)	95 % confidence interval (CI)	p-Value
**Socio-demographic core profile**			
**Age**	**1.02**	**[1.004, 1.04]**	**0.034**
**Male gender**	1.21	[0.70, 2.20]	0.380
**Race/Hispanic origin**	–	–	0.018
*Mexican Americans and Hispanics*	1.67	[0.78, 3.62]	–
*Non-Hispanic White*	2.54	[1.15, 5.56]	–
*Non-Hispanic Black*	2.45	[1.29, 4.68]	–
*Asians and other races*	1.00 (ref)	–	–
**Marital status**	–	–	0.720
*Married or living with partner*	1.06	[0.74, 1.53]	–
*Widowed, divorced or separated*	1.00 (ref)	–	–
**Educational attainment**	**–**	**–**	**<0.001**
Less than high school	1.59	[1.20, 2.10]	–
High school graduate	0.73	[0.38, 1.41]	–
College graduate or more	1.00 (ref)	–	–
**Annual household income**	–	–	**0.002**
Less than $45,000/yr	1.73	[0.88, 3.39]	–
$45,000 – Less than $75,000/yr	0.57	[0.24, 1.36]	–
More than $75,000/yr	1.00 (ref)	–	–
**Biochemical and hematologic parameters**			
**Fatty acids (model 1)**			
Percentage of saturated fatty acids	0.88	[0.80, 0.96]	**0.006**
Percentage of Omega-3 fatty acids	1.13	[0.89, 1.44]	0.290
**Fatty acids (model 2)**			
Percentage of monounsaturated fatty acids	1.14	[1.04, 1.25]	**0.006**
Percentage of Omega-3 fatty acids	1.29	[0.98, 1.70]	0.064
**Liver & kidney biomarkers**			
Albumin	0.41	[0.18, 0.98]	**0.044**
ALT	1.005	[0.99, 1.01]	0.190
Alkaline phosphatase	1.001	[0.99, 1.01]	0.390
Chloride	0.94	[0.86, 1.02]	0.100
Potassium	1.36	[0.60, 3.05]	0.440
Creatinine	1.25	[0.84, 1.85]	0.250
Glucose	0.99	[0.99, 1.01]	0.640
**Nutritional markers & vitamins**			
RBC folate	1.001	[0.99, 1.004]	0.280
5-Methyl tetrahydrofolate	0.97	[0.93, 1.004]	0.081
Serum selenium	1.01	[0.99, 1.02]	0.440
Serum copper	1.02	[1.01, 1.03]	**0.006**
Serum iron	1.006	[0.99, 1.02]	0.270
Blood mercury	0.90	[0.68, 1.19]	0.430
Total cholesterol	0.99	[0.98, 1.003]	0.180
**Hematologic CBC indices**			
WBC count	1.08	[0.96, 1.21]	0.220
Monocyte count	0.92	[0.47, 1.77]	0.780

All models are design-adjusted using Complex Samples Logistic Regression (CSLOGISTIC) commands to integrate survey design adjustments (weights WTMEC2YR, PSUs, and stratification). For multi-categorical blocks (Race, Marital Status, Education, Income), the p-value listed on the primary factor header denotes the overall multi-degree-of-freedom omnibus test significance. Fatty acid models are run independently due to composition collinearity: Model 1 adjusts for Saturated and Omega-3 profiles; Model 2 adjusts for Monounsaturated and Omega-3 profiles. All continuous biochemical covariates are adjusted per single-unit step increases. Ref, Reference baseline category. Bold text highlights statistical significance at p<0.05. Values in bold indicate those that were statistically significant.

### Biochemical, hepatic, and renal biomarkers

Serum albumin was the only variable in this group that had a significant inverse association with taste problems (p=0.044), i.e., higher albumin concentrations were associated with lower odds of taste dysfunction. A 1  g/dL increase in albumin reduced the odds of taste problems by 59 % (aOR=0.41, 95 % CI: [0.18, 0.98]). Other markers such as ALT (p=0.190), alkaline phosphatase (p=0.390), potassium (p=0.440), chloride (p=0.100), creatinine (p=0.250), and glucose (p=0.640), failed to reach statistical significance.

### Nutritional markers, micronutrients, and hematology

Of the heavy metals and nutritional factors tracked, serum copper remained associated with taste impairment (p=0.006). For each 1 µg/dL increase in copper concentration, the adjusted odds of taste dysfunction rose by 2 % (aOR=1.02, 95 % CI: [1.01, 1.03]).

The folate intermediate 5-methyl tetrahydrofolate showed a marginally significant association with taste dysfunction (aOR=0.97, 95 % CI: [0.93, 1.004]; p=0.081). No associations were observed between RBC folate (p=0.280), selenium (p=0.440), iron (p=0.270), mercury (p=0.430), total cholesterol (p=0.180), or complete blood count indices, including white blood cell (p=0.220) and monocyte (p=0.780) counts and taste problems.

## Discussion

This study examined the associations between demographic characteristics, biochemical and hematologic marker levels, and the odds of taste problems. Age, race, specifically, Non-Hispanic Whites and Black race, less than a high school education, and household income were sociodemographic factors associated with taste problems. Among biochemical markers, serum albumin, percentage of monounsaturated and saturated fatty acids, and serum copper levels were associated with taste problems. Hematologic markers were not associated with self-reported taste problems.

Increasing age has been widely described as being associated with taste problems. A study using NHANES 2011–2012 described that the prevalence of self-reported taste problems increased from approximately 19 % in adults aged 40 and older to 27 % in those aged 80 and older [1]. This age-related decline in gustatory function, termed presbygeusia, appears to be a physiological phenomenon that occurs even in the absence of comorbidities or medication effects [3], 4].

Several factors have been described that may contribute to age-related taste alterations. These include structural changes in taste buds, reduced number and altered morphology of taste receptor cells, decrease in saliva composition and salivary flow, which can lead to dry mouth, changes in the central nervous system, including age-related neuronal decline affecting gustatory processing, and dysbiosis of the oral microbiome. Other important contributors include polypharmacy, zinc deficiency, and oral/systemic diseases [1].

Objective testing confirms that individuals older than 60 years have significantly higher taste thresholds, with the most pronounced increases in detection thresholds occurring for sour and bitter tastes [14]. Taste loss in older adults has been linked to poor quality of life due to reduced appetite, nutritional imbalance, increased susceptibility to diseases, and diminished quality of life [15]. Further, smell and taste disorders can also serve as early signs of neurodegenerative disease, including dementia and Parkinson’s disease, and are associated with increased mortality [3], 4].

The association between race and taste problems is complex. Non-Hispanic White and Black populations have been reported to have a greater genetic diversity in bitter taste receptor genes (TAS2R38), which may contribute to higher odds of taste problems in them. Other risk factors like diet (heavy alcohol use), smoking, polypharmacy, and cardiovascular disease can lead to taste impairment in this group [16], [17], [18], [19], [20]. Lower education levels and annual household income are associated with higher smoking rates, poor nutrition, and greater occupational chemical exposures, which can in turn cause dysgeusia [2], 18], [21], [22], [23]. Both factors have previously been reported as independently associated with self-reported taste problems in population-based studies. In an NHANES 2011–2012 analysis of adults aged 40 and older, lower education was an independent risk factor for self-reported taste alteration after adjusting for health behaviors and chemosensory-related conditions, while family income at or below 110 % of the poverty threshold was an independent predictor of smell alteration [1]. A subsequent NHANES 2013–2014 cross-sectional study confirmed that low educational attainment and low family income were independently associated with higher prevalence of chemosensory impairment, and a JAMA Otolaryngology study of 7,340 NHANES participants further corroborated that individuals with subjective perception of taste loss had lower education levels and family income compared to those without taste loss [23], 24].

Several interconnected pathways likely underlie these associations. Lower socioeconomic status is linked to a greater burden of chronic diseases and polypharmacy, both established causes of taste dysfunction, with drug use and zinc deficiency accounting for approximately 21.7 % and 14.5 % of taste disorders, respectively [2], 25]. Higher prevalence of oral diseases such as dental caries, periodontal disease, and untreated oral pathology in this group of individuals may impair gustatory function [26]. Individuals with lower education are also more likely to be exposed to environmental toxins such as cadmium and lead through occupational settings, and higher blood cadmium levels have been linked to increased risk of chemosensory impairment, although we did not observe a significant association with blood cadmium levels [27]. Higher smoking rates among lower socioeconomic groups further contribute, as tobacco smoke directly damages taste receptor cells [22], 27]. Finally, reduced healthcare access may limit evaluation and management of taste complaints, as only approximately 20 % of the estimated 19.4 million Americans with smell and/or taste abnormalities discuss the problem with a healthcare provider [22].

The mechanism by which lower levels of saturated fatty acids cause taste problems likely involves reduced stimulation of lingual fat taste receptors (CD36 and GPR120), whose expression is directly regulated by levels of dietary fat. Low saturated fatty acid levels can also lead to deficiency of fat-soluble vitamins essential for taste bud cell membrane integrity and receptor function [28], [29], [30], [31]. Conversely, higher levels of monounsaturated fatty acids may lead to dysgeusia through chronic overstimulation and resulting downregulation of the lingual fat taste receptors. This, in turn, can reduce Ca2+ signaling in taste bud cells, blunting taste sensation [32], [33], [34]. Additionally, excessive Monounsaturated fatty acid levels can potentially alter the lipid raft composition of taste bud cell membranes. This can affect fat receptor trafficking and disrupt downstream ERK1/2-MAPK signaling cascades that relay taste signals from the lingual taste receptors to the sensory nerve endings [29], 35].

Albumin deficiency-related taste dysfunction is likely due to the fact that albumin is a carrier for zinc, which is critical for taste bud cell turnover. Therefore, hypoalbuminemia reduces zinc bioavailability and raises taste detection thresholds [36]. Additionally, low albumin reflects underlying protein-energy malnutrition and systemic inflammation, which both independently impair taste bud regeneration and salivary gland function [37], [38], [39]. Finally, elevated serum copper negatively impacts zinc bioavailability, which imbalances the Cu/Zn ratio, in turn, lower zinc levels are associated with increased taste recognition thresholds [40]. High copper levels also generate reactive oxygen species that damage taste bud cells and salivary proteins that are crucial for taste [41], 42].

### Clinical and research implications

The findings highlight the importance of routine taste dysfunction screening, especially in older adults, Non-Hispanic White and Black individuals, and those with lower education and annual income. They also suggest that measurement of serum fatty acid concentration, serum albumin, and copper levels may aid in understanding the pathophysiology of dysgeusia. Addressing modifiable risk factors such as polypharmacy, smoking, occupational chemical exposures, and limited access to dental and medical care in underserved populations may reduce the prevalence of taste dysfunction . As our study was retrospective, causal inferences cannot be drawn from it. Future prospective research studies are needed to investigate whether the findings of this study are causally linked to taste dysfunction, particularly stratified by severity and evidence of objective taste abnormalities. In addition to that, studies should examine the role of sociodemographic factors to see if they are causally linked to taste dysfunction.

### Strengths and limitations

This study had several strengths. One of these was the use of NHANES data, providing a large, nationally representative sample, promoting the generalization of the findings to the U.S. Population. Another strength was that including both demographic characteristics and biochemical markers made the study comprehensive in the exploration of factors associated with taste dysfunction.

However, several limitations should also be recognized. The observational design of this study does not allow us to establish directionality, meaning that taste dysfunction may itself alter dietary intake and nutritional status, rather than solely resulting from them. Further, taste problems were self-reported which lends itself to recall bias. Also, people might have experienced mild taste loss that wasn’t reported and wouldn’t have been recognized without objective testing methods, like whole-mouth gustatory testing. Finally, while the analysis was adjusted for many covariates, there were many unmeasured factors, such as medication, oral hygiene status, smoking history, dietary intake, BMI, height and weight. These could be incorporated in future studies to further adjust the analysis and improve accuracy.

## Conclusions

In conclusion, this study found that certain biochemical markers were independently associated with taste problems after adjusting for demographic characteristics; partially supporting the study hypothesis. Hematological markers however, were not independently associated. Specifically, lower saturated fatty acid and albumin levels, as well as higher monounsaturated fatty acid and copper levels, were independently associated with taste dysfunction. Among sociodemographic factors, older age, Non-Hispanic White and Black race, lower educational attainment, and lower annual household income were all independently associated. These findings underscore the multifactorial nature of taste dysfunction and highlight the need for larger prospective studies to elucidate causal pathways and determine whether modifying biochemical factors can improve taste dysfunction.

## Supplementary Material

Supplementary Material
