# Supplementary material for: Sociodemographic and biochemical correlates of self-reported taste dysfunction: an NHANES analysis
Source: Open Med (Wars). 2026 Aug 24;21(1):20261496. doi: 10.1515/med-2026-1496 (PMC13499027; doi:10.1515/med-2026-1496)
Supplement: Supplementary file 1 [file j_med-2026-1496_suppl_001.docx]

**Supplementary Table 1. Comprehensive Design-Adjusted Serum Biochemical, Hematological, and Fatty Acid Profiling Stratified by 12-Month Self-Reported Taste Dysfunction: NHANES 2011–2014**

| **Laboratory Biomarker** | **With Taste Problems**  **EMM (SE)** | **Without Taste Problems**  **EMM (SE)** | **P-Value** |
| --- | --- | --- | --- |
| **Metabolic, Liver & Kidney Biomarkers** |  |  |  |
| Albumin (g/dL) | 4.15 (0.04) | 4.27 (0.01) | **0.013** |
| Alanine Aminotransferase ALT (IU/L) | 31.5 (6.4) | 24.7 (0.3) | 0.300 |
| Aspartate Aminotransferase AST (IU/L) | 29.9 (3.2) | 26.3 (0.4) | 0.280 |
| Alkaline Phosphatase (IU/L) | 75.5 (2.5) | 68.0 (0.6) | **0.009** |
| Blood Urea Nitrogen (mg/dL) | 14.6 (0.8) | 13.9 (0.1) | 0.400 |
| Total Calcium (mg/dL) | 9.30 (0.06) | 9.40 (0.02) | 0.440 |
| Creatine Phosphokinase CPK (IU/L) | 142.5 (23.6) | 133.0 (3.6) | 0.710 |
| Bicarbonate (mmol/L) | 25.4 (0.2) | 25.1 (0.1) | 0.210 |
| Creatinine (mg/dL) | 0.99 (0.04) | 0.91 (0.01) | **0.030** |
| Gamma Glutamyl Transferase GGT (IU/L) | 30.0 (2.5) | 29.7 (1.4) | 0.930 |
| Serum Glucose (mg/dL) | 113.5 (6.3) | 103.1 (1.1) | 0.110 |
| Lactate Dehydrogenase LDH (IU/L) | 135.9 (2.8) | 129.5 (0.9) | **0.042** |
| Phosphorus (mg/dL) | 3.74 (0.08) | 3.71 (0.01) | 0.650 |
| Total Bilirubin (mg/dL) | 0.69 (0.03) | 0.71 (0.02) | 0.530 |
| Total Protein (g/dL) | 6.90 (0.06) | 7.04 (0.20) | 0.340 |
| Uric Acid (mg/dL) | 5.60 (0.15) | 5.50 (0.06) | 0.380 |
| Sodium (mmol/L) | 138.6 (0.2) | 138.9 (0.1) | 0.170 |
| Potassium (mmol/L) | 4.01 (0.03) | 3.90 (0.01) | 0.170 |
| Chloride (mmol/L) | 102.9 (0.2) | 103.8 (0.1) | **0.008** |
| Osmolality (mmol/Kg) | 278.2 (0.50) | 278.0 (0.19) | 0.600 |
| Globulin (g/dL) | 2.80 (0.06) | 2.80 (0.02) | 0.290 |
| **Environmental Heavy Metals & Trace Minerals** |  |  |  |
| Blood Cadmium (µg/dL) | 0.55 (0.03) | 0.56 (0.02) | 0.800 |
| Blood Lead (µg/dL) | 1.70 (0.09) | 1.70 (0.12) | 0.660 |
| Blood Mercury, total (µg/dL) | 1.45 (0.13) | 1.75 (0.22) | 0.210 |
| Blood Selenium (µg/dL) | 203.2 (8.9) | 194.7 (1.7) | 0.340 |
| Blood Manganese (µg/dL) | 9.7 (0.4) | 9.5 (0.1) | 0.520 |
| Serum Copper (µg/dL) | 124.3 (3.6) | 116.6 (1.4) | **0.046** |
| Serum Selenium (µg/dL) | 133.3 (3.7) | 130.9 (1.6) | 0.570 |
| Serum Zinc (µg/dL) | 81.2 (2.7) | 83.2 (0.6) | 0.440 |
| Iron, refrigerated (µg/dL) | 79.9 (3.4) | 87.5 (1.2) | 0.065 |
| **Circulating Serum Lipid Homeostasis** |  |  |  |
| Direct HDL-Cholesterol (mg/dL) | 50.9 (1.6) | 53.9 (0.6) | 0.090 |
| Triglycerides (mg/dL) | 175.6 (8.5) | 164.2 (4.2) | 0.290 |
| LDL-Cholesterol (mg/dL) | 110.4 (7.7) | 118.8 (1.1) | 0.290 |
| Total Cholesterol (mg/dL) | 191.8 (6.0) | 199.9 (1.3) | 0.240 |
| **Hematologic Indices (CBC Counts)** |  |  |  |
| White Blood Cell Count (10^3^ cells/µL) | 7.2 (0.2) | 6.9 (0.1) | 0.230 |
| Lymphocyte Number (10^3^ cells/µL) | 2.0 (0.1) | 1.9 (0.03) | 0.550 |
| Monocyte Number (10^3^ cells/µL) | 0.56 (0.03) | 0.52 (0.14) | 0.150 |
| Segmented Neutrophils (10^3^ cells/µL) | 4.4 (0.2) | 4.2 (0.1) | 0.350 |
| Eosinophils Number (10^3^ cells/µL) | 0.20 (0.01) | 0.20 (0.01) | 0.770 |
| Basophils Number (10^3^ cells/µL) | 0.04 (0.01) | 0.04 (0.002) | 0.740 |
| Red Blood Cell Count (10^6^cells/µL) | 4.5 (0.08) | 4.5 (0.02) | 0.710 |
| Hemoglobin (g/dL) | 13.9 (0.2) | 14.1 (0.1) | 0.340 |
| MCHC (g/dL) | 34.3 (0.1) | 34.4 (0.1) | 0.770 |
| Platelet Count (10^3^ cells/µL) | 231.1 (4.9) | 233.7 (2.0) | 0.590 |
| **Serum Folates & Vitamin Co-Factors** |  |  |  |
| RBC Folate (ng/mL) | 620.9 (33.9) | 546.7 (11.4) | **0.038** |
| 5-Methyl-tetrahydrofolate (nmol/L) | 48.3 (3.9) | 44.3 (0.9) | 0.280 |
| 5-Formyl-tetrahydrofolate (nmol/L) | 0.22 (0.01) | 0.26 (0.03) | 0.190 |
| 5,10-Methenyl-tetrahydrofolate (nmol/L) | 0.27 (0.02) | 0.26 (0.004) | 0.440 |
| Vitamin B12 (pg/mL) | 661.8 (44.7) | 640.6 (15.2) | 0.640 |
| Total 25OHD2 + 25OHD3 (nmol/L) | 73.8 (3.1) | 75.3 (1.8) | 0.680 |
| 25OHD2 (nmol/L) | 5.4 (1.0) | 4.9 (0.7) | 0.620 |
| 25OHD3 (nmol/L) | 68.4 (7.8) | 70.4 (1.9) | 0.510 |
| epi-25OHD3 (nmol/L) | 4.4 (0.51) | 4.7 (0.26) | 0.520 |
| **Circulating Serum Fatty Acid Speciation Profiles** |  |  |  |
| Capric Acid (C10:0) (µmol/L) | 3.35 (0.810) | 3.35 (0.245) | 1.000 |
| Lauric Acid (C12:0) (µmol/L) | 18.2 (2.7) | 15.8 (0.7) | 0.450 |
| Myristic Acid (14:0) (µmol/L) | 182.1 (15.7) | 159.1 (5.1) | 0.210 |
| Pentadecanoic Acid (C15:0) (µmol/L) | 31.1 (1.9) | 26.6 (0.6) | **0.035** |
| Palmitic Acid (16:0) (µmol/L) | 3506.1 (196.5) | 3209.5 (67.4) | 0.180 |
| Margaric Acid (C17:0) (µmol/L) | 37.0 (1.7) | 33.5 (0.7) | 0.078 |
| Stearic Acid (18:0) (µmol/L) | 767.6 (31.7) | 753.7 (13.9) | 0.740 |
| Arachidic Acid (20:0) (µmol/L) | 24.4 (1.1) | 25.3 (0.4) | 0.450 |
| Docosanoic Acid (22:0) (µmol/L) | 64.7 (2.6) | 70.8 (0.9) | **0.030** |
| Tricosanoic Acid (C23:0) (µmol/L) | 28.5 (1.7) | 30.3 (0.3) | 0.280 |
| Lignoceric Acid (24:0) (µmol/L) | 54.2 (2.4) | 60.2 (0.8) | **0.022** |
| Myristoleic Acid (14:1n-5) (µmol/L) | 12.7 (1.6) | 10.4 (0.4) | 0.230 |
| Palmitoleic Acid (16:1n-7) (µmol/L) | 349.2 (35.8) | 293.3 (8.6) | 0.180 |
| cis-Vaccenic Acid (18:1n-7) (µmol/L) | 180.2 (9.5) | 166.1 (3.2) | 0.200 |
| Oleic Acid (18:1n-9) (µmol/L) | 2853.1 (167.1) | 2502.5 (68.6) | 0.070 |
| Eicosenoic Acid (20:1n-9) (µmol/L) | 16.9 (1.2) | 16.2 (0.5) | 0.530 |
| Nervonic Acid (24:1n-9) (µmol/L) | 86.7 (5.5) | 89.9 (1.0) | 0.590 |
| Linoleic Acid (18:2n-6) (µmol/L) | 3777.1 (138.5) | 3940.0 (59.2) | 0.320 |
| alpha-Linolenic Acid (18:3n-3) (µmol/L) | 108.4 (8.7) | 97.5 (2.3) | 0.230 |
| gamma-Linolenic Acid (18:3n-6) (µmol/L) | 76.9 (5.8) | 69.3 (1.9) | 0.290 |
| Stearidonic Acid (C18:4n-3) (µmol/L) | 4.8 (0.4) | 4.7 (0.2) | 0.780 |
| Eicosadienoic Acid (20:2n-6) (µmol/L) | 25.9 (1.9) | 24.8 (0.5) | 0.570 |
| homo-gamma-Linolenic Acid (20:3n-6) (µmol/L) | 181.5 (12.0) | 171.8 (3.6) | 0.480 |
| Eicosatrienoic Acid (C20:3n-9) (µmol/L) | 10.8 (1.1) | 9.3 (0.2) | 0.230 |
| Arachidonic Acid (20:4n-6) (µmol/L) | 931.9 (34.1) | 928.3 (13.5) | 0.940 |
| Eicosapentaenoic Acid (20:5n-3) (µmol/L) | 111.9 (35.8) | 80.9 (3.2) | 0.400 |
| Docosatetraenoic Acid (22:4n-6) (µmol/L) | 31.5 (1.9) | 27.9 (0.7) | 0.130 |
| Docosapentaenoic Acid (22:5n-3) (µmol/L) | 65.7 (3.9) | 57.7 (0.9) | 0.070 |
| Docosapentaenoic Acid (22:5n-6) (µmol/L) | 23.4 (1.7) | 20.7 (0.5) | 0.130 |
| Docosahexaenoic Acid (22:6n-3) (µmol/L) | 178.1 (12.6) | 174.7 (5.6) | 0.830 |

Footnote. Continuous biomarker values are presented as Estimated Marginal Means (EMM) with corresponding Standard Errors (SE), Reference ranges correspond to standard clinical assay specifications documented by the NHANES Laboratory Sciences Division. Boldface indicates statistically significant differences at p < 0.05. **Acronym expansions:** ALT = Alanine Aminotransferase; AST = Aspartate Aminotransferase; ALP = Alkaline Phosphatase; BUN = Blood Urea Nitrogen; CPK = Creatine Phosphokinase; GGT = Gamma‑Glutamyl Transferase; LDH = Lactate Dehydrogenase; HDL = High‑Density Lipoprotein; LDL = Low‑Density Lipoprotein; RBC = Red Blood Cell; WBC = White Blood Cell; MCHC = Mean Corpuscular Hemoglobin Concentration; 25OHD2 = 25‑Hydroxyvitamin D2; 25OHD3 = 25‑Hydroxyvitamin D3; epi‑25OHD3 = Epimeric 25‑Hydroxyvitamin D3.
